# Translational Assessment of a Cell-Penetrating Peptide Topical Formulation for Repairing Barrier Dysfunction in Human Skin

**DOI:** 10.3390/ijms27146357

**Published:** 2026-07-17

**Authors:** Young In Lee, Ahlim Min, Wooram Kim, Jinyoung Jung, Hosung Choi, Jewan Kaiser Hwang, Ngoc Ha Nguyen, Inhee Jung, Hyojin Roh

**Affiliations:** 1 Department of Dermatology & Cutaneous Biology Research Institute, Yonsei University College of Medicine, Seoul 03722, Republic of Korea; ylee1124@yuhs.ac (Y.I.L.); nguyenngocha7996@gmail.com (N.H.N.); 2 Scar Laser and Plastic Surgery Center, Yonsei Cancer Hospital, Seoul 03722, Republic of Korea; 3 Min&Min Clinic, Seoul 06124, Republic of Korea; drminarim@naver.com; 4 Wyne Plastic Surgery Clinic, Cheongju 28540, Republic of Korea; hezit@hanmail.net; 5 Mymirae Dermatologic Clinic, Seoul 07326, Republic of Korea; yoracle@naver.com; 6 PIENA Aesthetic Medical, Seoul 06136, Republic of Korea; uroskin2121@gmail.com; 7 Mymirae Research Institute for Dermatologic Science, Seoul 07326, Republic of Korea; kaiserwan@hanmail.net; 8 Department of Dermatology, University of Medicine and Pharmacy at Ho Chi Minh City, Ho Chi Minh City 70000, Vietnam; 9 Global Medical Research Center Co., Ltd., Seoul 06526, Republic of Korea; ihjung@gmrc.co.kr

**Keywords:** cell-penetrating peptides, skin barrier, pruritus, inflammation, keratinocytes, filaggrin proteins, macrophages, leukocytes, ultraviolet B

## Abstract

Skin barrier dysfunction and pruritus, central to inflammatory dermatoses, are closely associated with impaired epidermal structural proteins and persistent inflammatory signaling. To address these pathological features, this study aimed to explore the potential anti-inflammatory, barrier-restorative, and anti-pruritic effects of DualPep-ATO, a human-derived cell-penetrating peptide (CPP), using integrated in vitro, ex vivo, and exploratory clinical approaches. Allergic and inflammatory responses were evaluated in rat basophilic leukemia cells and lipopolysaccharide-stimulated macrophages, while barrier-repair properties were partially assessed in ultraviolet B (UVB)-irradiated human epidermal keratinocytes and ex vivo human skin tissue. Mechanistically, DualPep-ATO exhibited no cytotoxicity and successfully suppressed β-hexosaminidase release, nitric oxide production, and pro-inflammatory cytokine expression. Furthermore, it contributed to mitigating skin barrier damage by restoring key epidermal structural proteins, specifically filaggrin, loricrin, and involucrin. Translating these findings clinically, a four-week topical application of a DualPep-ATO cream in adults with compromised skin significantly decreased transepidermal water loss and itch severity. With high participant satisfaction and no reported adverse events, this peptide may serve as a promising peptide-based topical strategy for inflammatory skin conditions associated with barrier dysfunction and pruritus.

## 1. Introduction

The skin barrier plays an integral part in safeguarding the body against external insults and microbes. However, its integrity can be easily compromised by a variety of inflammatory and allergic skin conditions, in which pro-inflammatory cytokines degrade the epidermal structure and trigger pruritus and scratching behaviors [1,2,3,4,5]. Histologically, the loss of structural proteins, such as involucrin, loricrin, and filaggrin, accompanies a damaged skin barrier [6], increasing the vulnerability towards extrinsic pathogens and pruritogens, thereby exacerbating the pruritus and scratching to induce further damage to the skin [7]. This creates a vicious itch–scratch cycle that perpetuates barrier compromise [7]. Ultimately, this chronic loop severely impacts patients’ quality of life by disrupting sleep, impairing concentration, and diminishing mental well-being [8].

Conventional treatments to dampen local inflammatory responses, including topical corticosteroids, calcineurin inhibitors, and antihistamines, suppress immune activation and alleviate itching [9]. However, prolonged corticosteroid use can cause side effects such as skin thinning, telangiectasia, and barrier compromise, while calcineurin inhibitors may lead to burning sensations and increased infection risk [10]. Antihistamines provide temporary itch relief but fail to address underlying inflammation [11]. These treatments may fail to restore the skin barrier or prevent recurrence. On the other hand, moisturizers can heal the damaged skin barrier by supplying lipids or natural moisturizing factors, or prevent the loss of hydration by acting as an occludent [12]. Nevertheless, the employment of moisturizers in skin conditions with a compromised skin barrier may bring further damage due to their toxicity and not address the underlying abnormalities [13]. Recently, the development of bioactive peptides has demonstrated their potential in alleviating skin inflammation, but their low membrane permeability remains a significant challenge [14]. Furthermore, advanced biologic agents offer intensive options for systemic inflammatory skin diseases, yet they are frequently accompanied by a variety of immune-related adverse events. These disadvantages underscore the need for safer, complementary therapies that can suppress inflammation and enhance barrier recovery.

Cell-penetrating peptides (CPPs) are short amino acid sequences that can traverse cellular membranes and enable efficient intracellular delivery of therapeutic molecules [15,16,17]. Owing to their excellent permeability and low cytotoxicity, CPPs hold significant promise for transdermal applications in dermatology. For example, DualPep-Shine, a Neurog-1–derived peptide, effectively suppresses melanin synthesis in melanocytes, providing a safe approach for skin brightening [18]. Recently, peptides from the Speckled Protein (SP) family, identified through the Intra-Dermal Delivery Technology (IDDT) platform, demonstrated strong anti-inflammatory effects by inhibiting Nuclear Factor Kappa B (NF-κB) and Signal Transducer and Activator of Transcription 3 (STAT3) signaling while showing enhanced membrane affinity and sustained intracellular retention [19]. Collectively, we hypothesize that CPPs may represent promising therapeutic candidates for modulating inflammation and restoring skin barrier integrity. Topical application emerges as a particularly viable strategy as these peptides might penetrate the skin effectively while retaining their intrinsic biological activity. Nevertheless, the translational relevance of these effects under formulation-compatible and clinically applicable conditions remains insufficiently explored.

Hence, this study investigates the effects of DualPep-ATO, the experimently discovered CPP from the SP family, in reducing pruritus, allergic and inflammatory reactions, as well as helping restore the skin barrier. Our study integrated in vitro, ex vivo models, and an exploratory, hypothesis-generating, open-label study in adults with pruritus and impaired skin barrier to integrally assess DualPep-ATO’s efficacy.

## 2. Results

### 2.1. In Vitro Study

#### 2.1.1. Cell Viability

**Figure 1:**
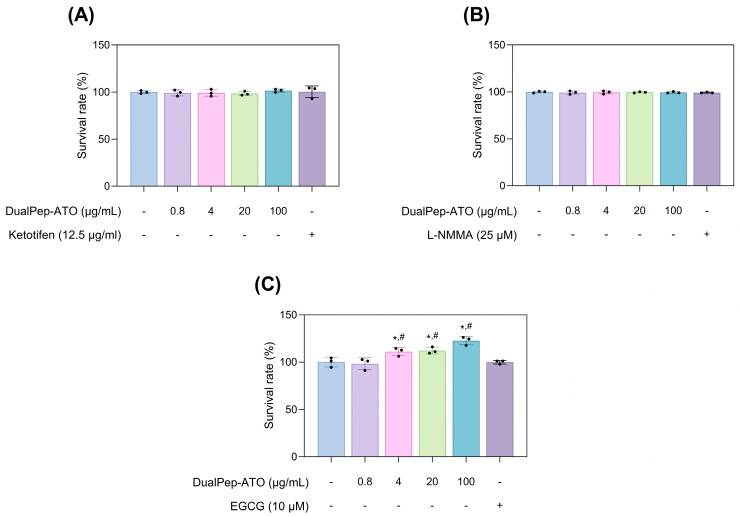
Effects of DualPep-ATO on cell viability and proliferation in immune and epidermal cells. (**A**) RBL-2H3, (**B**) RAW 264.7, and (**C**) HEKn cells were treated with increasing concentrations of DualPep-ATO (0.8–100 μg/mL) or the corresponding positive controls (ketotifen, 12.5 μg/mL; L-NMMA, 25 μM; and EGCG, 10 μM, respectively) for 24 h. DualPep-ATO did not induce cytotoxicity in RBL-2H3 or RAW 264.7, maintaining cell viability above 95% across all tested concentrations. In HEKn, it significantly enhanced cell proliferation at 4, 20, and 100 μg/mL compared with the untreated and positive controls. Data are presented as mean ± SD. * *p* < 0.05 vs. negative control. # *p* < 0.05 vs. positive control. L-NMMA, NG-methyl-L-arginine acetate salt; EGCG, epigallocatechin gallate.

Following DualPep-ATO treatment (0.8–100 µg/mL), rat basophil leukemia cells (RBL-2H3) and mouse macrophages (RAW 264.7) maintained viability above 95%, comparable to untreated and positive control groups (ketotifen and NG-methyl-L-arginine acetate salt (L-NMMA)) (Figure 1A,B). Meanwhile, human epidermal keratinocytes, neonatal (HEKn) exhibited significantly increased proliferation relative to the negative control (*p* < 0.05) and the positive control epigallocatechin gallate (EGCG) (*p* < 0.05) (Figure 1C). These findings demonstrate that DualPep-ATO is safe within the tested concentration range. We selected three highest concentrations for further experiments (4, 20, and 100 µg/mL) due to their favorable cell proliferation.

#### 2.1.2. Mitigation of Allergic Reaction via Decrease in β-Hexosaminidase (β-HEX) Secretion in Rat Basophil Leukemia Cells

**Figure 2:**
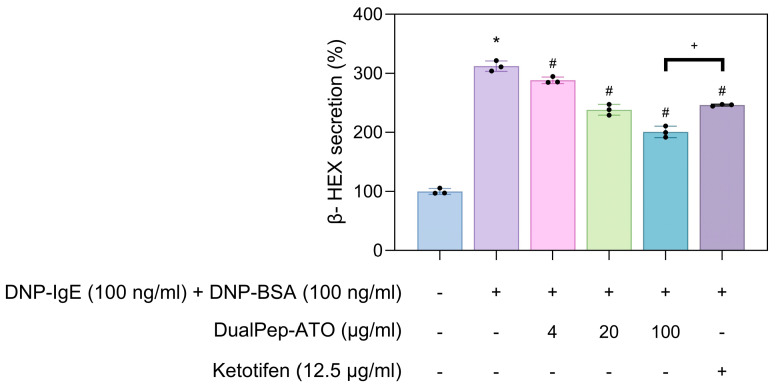
Inhibitory effect of DualPep-ATO on β-HEX secretion in RBL-2H3 cells. Cells were sensitized with DNP-IgE (100 ng/mL) and stimulated with DNP-BSA (100 ng/mL) to induce β-HEX release. Pretreatment with DualPep-ATO (4–100 µg/mL) suppressed β-HEX secretion in a dose-dependent manner. Ketotifen (12.5 µg/mL) was used as a positive control. Data are presented as mean ± SD. * *p* < 0.05 vs. control; # *p* < 0.05 vs. DNP-IgE + DNP-BSA group; + *p* < 0.05 vs. ketotifen group. DNP-IgE, anti-dinitrophenyl immunoglobulin E; DNP-BSA, 2,4-Dinitrophenylated Bovine Serum Albumin.

In allergy-mediated pruritus, mast cells release a variety of mediators that induce itch or signify their degranulation, including β-HEX [20]. In our experiment, RBL-2H3 cells were first sensitized with anti-dinitrophenyl immunoglobulin E (DNP-IgE), followed by stimulation with 2,4-Dinitrophenylated Bovine Serum Albumin (DNP-BSA), which increased their β-HEX secretion. This effect was attenuated by DualPep-ATO pretreatment in a dose-dependent manner (*p* < 0.05, Figure 2). At 100 µg/mL, DualPep-ATO showed a better inhibitory effect compared to that of ketotifen (*p* < 0.05).

#### 2.1.3. Anti-Inflammatory Effect on Mouse Macrophages

**Figure 3:**
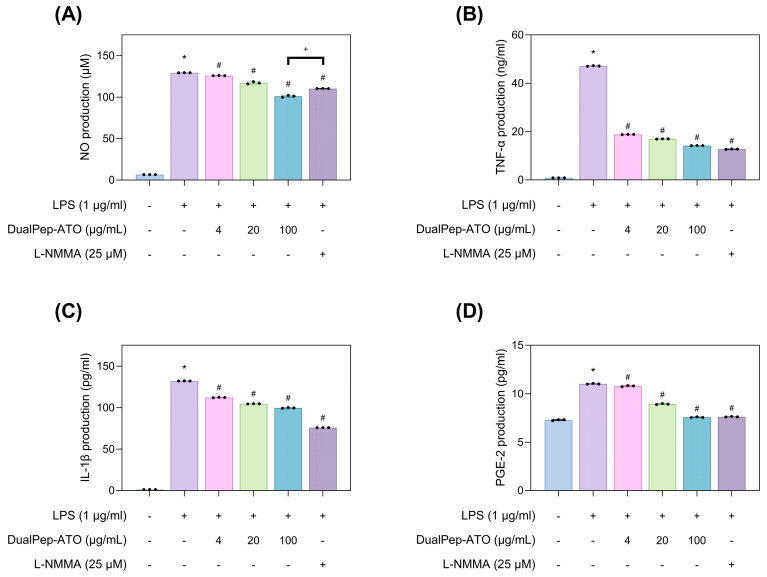
Anti-inflammatory effects of DualPep-ATO on LPS-stimulated RAW 264.7. Cells were treated in serum-free DMEM containing LPS to induce inflammation, followed by treatment with DualPep-ATO (4, 20, or 100 μg/mL) or the positive control L-NMMA (25 μM) for an additional 24 h. The production levels of (**A**) NO, (**B**) TNF-α, (**C**) IL-1β, and (**D**) PGE-2 were measured in the culture supernatants by ELISA. DualPep-ATO dose-dependently suppressed the production of key pro-inflammatory mediators. Data are presented as mean ± SD. * *p* < 0.05 vs. negative control; # *p* < 0.05 vs. LPS group; + *p* < 0.05 vs. L-NMMA group. NO, nitric oxide; TNF-α, tumor necrosis factor-alpha; IL, interleukin; PGE-2, prostaglandin E2; LPS, lipopolysaccharide; L-NMMA, NG-methyl-L-arginine acetate salt.

Activated macrophages, upon activation, release a host of inflammatory cytokines, including nitric oxide (NO), tumor necrosis factor-alpha (TNF-α), interleukin (IL)-1β, and prostaglandin E2 (PGE-2) [21,22,23]. In our RAW 264.7 cells, stimulation by lipopolysaccharide (LPS), a bacterial toxin, markedly increased the production of these inflammatory mediators (Figure 3A–D). Treatment with DualPep-ATO significantly suppressed the production of all four markers in a dose-dependent manner (*p* < 0.05). At 100 µg/mL, the inhibitory effects on NO were higher compared to those of the positive control L-NMMA (*p* < 0.05).

#### 2.1.4. Production of Skin Epidermal Barrier Proteins in Human Epidermal Keratinocytes

**Figure 4:**
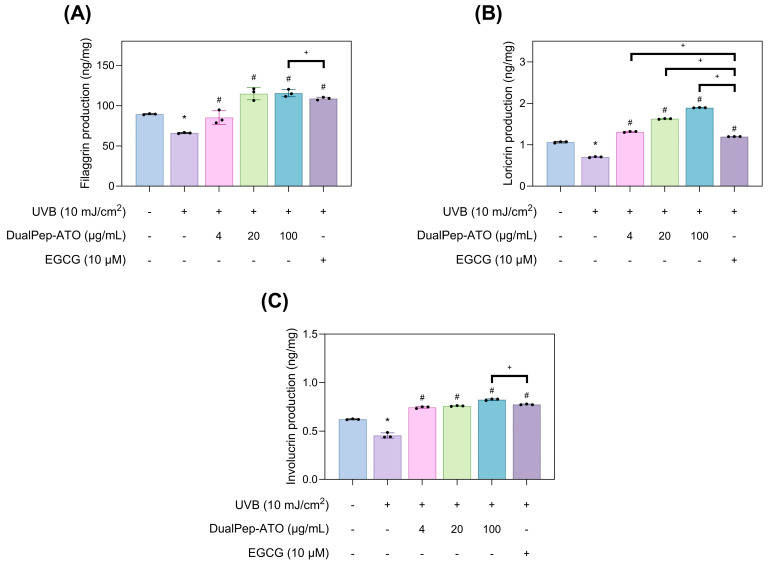
Restorative effects of DualPep-ATO on epidermal barrier protein production in UVB-irradiated HEKn. Cells were exposed to UVB irradiation (10 mJ/cm^2^), followed by treatment with increasing concentrations of DualPep-ATO (4–100 µg/mL) or the positive control EGCG (10 µM) for 24 h. Production levels of (**A**) filaggrin, (**B**) loricrin, and (**C**) involucrin were measured in the culture supernatants by ELISA. DualPep-ATO dose-dependently restored the UVB-induced suppression of skin barrier-related proteins. Data are presented as mean ± SD. * *p* < 0.05 vs. negative control; # *p* < 0.05 vs. UVB-irradiated group; + *p* < 0.05 vs. EGCG group. UVB, ultraviolet B; EGCG, epigallocatechin gallate.

UVB irradiation significantly reduced the production of key epidermal barrier proteins, including filaggrin, loricrin, and involucrin, in HEKn (*p* < 0.05, Figure 4A–C). Treatment with DualPep-ATO effectively restored the levels of all three proteins in a dose-dependent manner (*p* < 0.05). In particular, the 100 µg/mL group showed the greatest recovery, greater than that of the positive control EGCG (10 µM) for filaggrin (*p* < 0.05), loricrin (*p* < 0.05), and involucrin (*p* < 0.05).

### 2.2. Ex Vivo Study

#### 2.2.1. Anti-Inflammatory Effect on Human Skin Tissue

**Figure 5:**
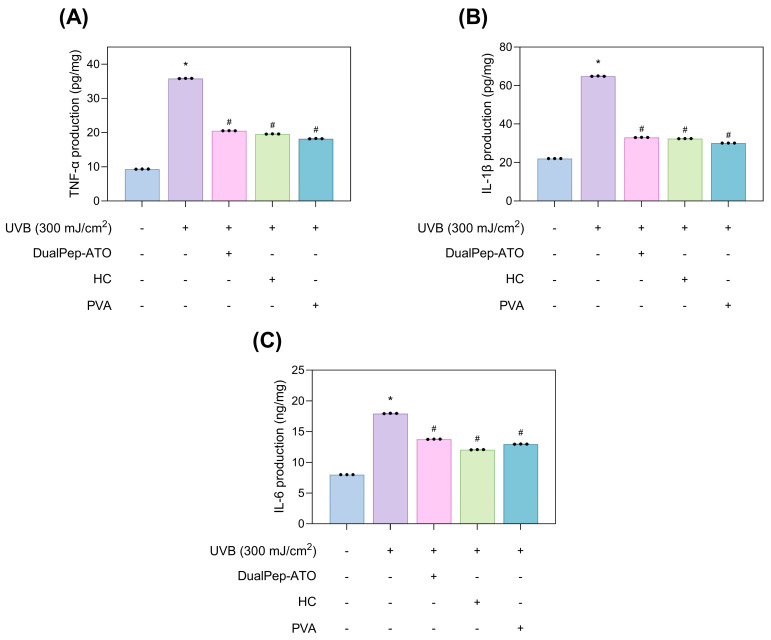
Anti-inflammatory effects of DualPep-ATO on UVB-irradiated human skin tissue. Human skin tissues were exposed to UVB irradiation (300 mJ/cm^2^), before being treated with DualPep-ATO or the positive control HC or PVA for 24 h. Production levels of (**A**) TNF-α, (**B**) IL-1β, and (**C**) IL-6 were quantified in tissue supernatants by ELISA. UVB irradiation markedly elevated pro-inflammatory cytokine expression, which was counteracted by DualPep-ATO. Data are presented as mean ± SD. * *p* < 0.05 vs. control; # *p* < 0.05 vs. UVB-irradiated group. UVB, ultraviolet B; TNF-α, tumor necrosis factor-alpha; IL, interleukin; HC, hydrocortisone; PVA, prednisolone valeroacetate.

UVB irradiation markedly increased the production of inflammatory cytokines TNF-α, IL-1β, and IL-6 in human skin tissue, and this effect was reversed by treatment with DualPep-ATO (*p* < 0.05, Figure 5A–C). Furthermore, the impact of DualPep-ATO was almost comparable to that of hydrocortisone (HC, *p* < 0.05) and prednisolone valeroacetate (PVA, *p* < 0.05). These results demonstrate that DualPep-ATO effectively mitigates UVB-induced inflammation in human skin tissue by reducing the expression of pro-inflammatory cytokines.

#### 2.2.2. Production and Expression of Skin Epidermal Barrier Proteins in Human Skin Tissue

**Figure 6:**
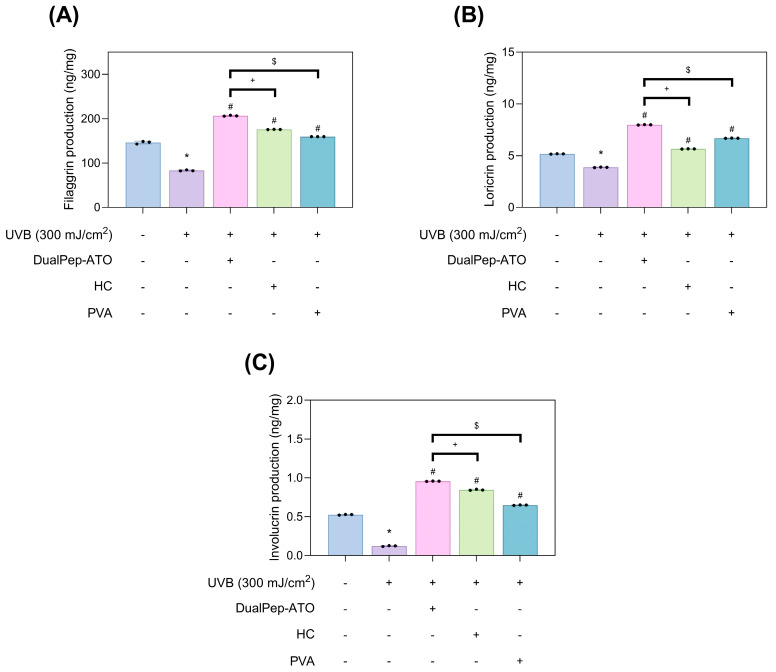
Restorative effects of DualPep-ATO on skin barrier-related protein production in UVB-irradiated human skin tissue. The tissues were exposed to UVB irradiation (300 mJ/cm^2^), before being treated with DualPep-ATO or the positive control HC or PVA for 24 h. Production levels of (**A**) filaggrin, (**B**) loricrin, and (**C**) involucrin were quantified in tissue supernatants by ELISA. UVB irradiation significantly suppresses the production of these proteins, which was reversed by DualPep-ATO. Data are presented as mean ± SD. * *p* < 0.05 vs. control; # *p* < 0.05 vs. UVB-irradiated group; + *p* < 0.05 vs. HC-treated group; $ *p* < 0.05 vs. PVA-treated group. UVB, ultraviolet B; HC, hydrocortisone; PVA, prednisolone valeroacetate.

UVB irradiation significantly reduced the production of major epidermal barrier proteins, including filaggrin, loricrin, and involucrin (*p* < 0.05, Figure 6A–C). Treatment with DualPep-ATO markedly restored their levels, higher than those of the positive controls HC (*p* < 0.05) and PVA (*p* < 0.05).

**Figure 7:**
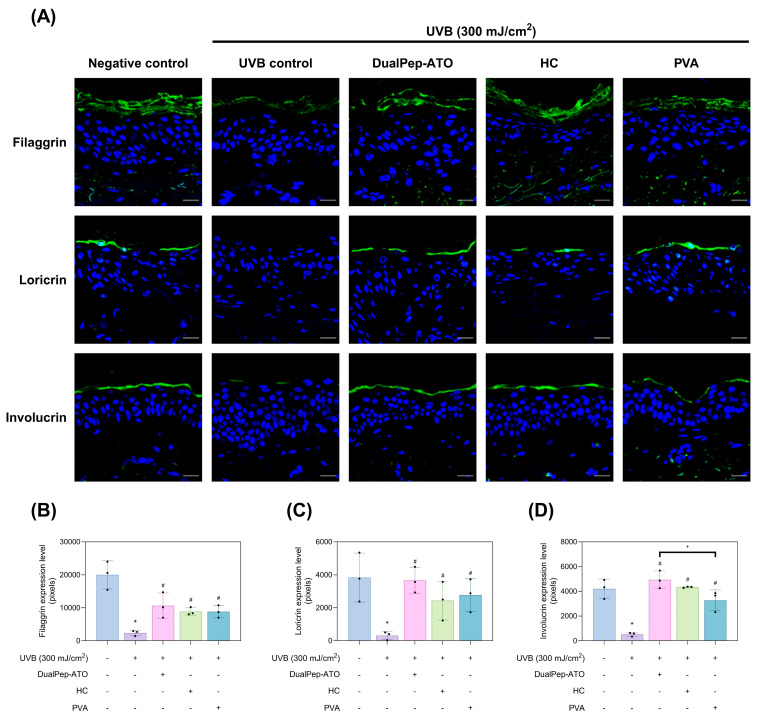
Protective effects of DualPep-ATO on skin barrier-related protein expression. Human skin tissues were exposed to UVB irradiation (300 mJ/cm^2^) and subsequently treated with DualPep-ATO, positive control HC or PVA for 24 h. Immunofluorescence and quantitative intensity analysis demonstrate that DualPep-ATO counteracts the UVB-induced degradation of these proteins. (**A**) Representative IF images showing the distribution and expression of filaggrin, loricrin, and involucrin (green) in human skin tissue sections. Nuclei were counterstained with DAPI (blue). Scale bar = 100 μm. (**B**–**D**) Quantitative evaluation of fluorescence intensity (pixels) for filaggrin (**B**), loricrin (**C**), and involucrin (**D**) corresponding to panel (**A**). HC and PVA served as positive controls. Data are presented as mean ± SD. * *p* < 0.05 vs. control; # *p* < 0.05 vs. UVB-irradiated group; + *p* < 0.05 vs. PVA group. DAPI, 4′,6-Diamidino-2-Phenylindole; UVB, ultraviolet B; HC, hydrocortisone; PVA, prednisolone valeroacetate.

Immunofluorescence (IF) analysis further confirmed these findings, showing visibly increased fluorescence intensity of filaggrin, loricrin, and involucrin in DualPep-ATO-treated skin compared with the UVB control (Figure 7A). Quantitative analysis demonstrated significantly higher expression levels of these proteins in the DualPep-ATO group (*p* < 0.05, Figure 7B–D). The upregulation in these proteins by DualPep-ATO, especially involucrin, was higher compared to those of the comparator PVA (*p* < 0.05).

### 2.3. Clinical Study

Given the reproducible anti-inflammatory and barrier-supportive effects of DualPep-ATO in cellular and human skin tissue models, we next performed an exploratory, hypothesis-generating clinical study to examine its potential relevance in humans with pruritic, barrier-impaired skin. Our clinical study recruited 21 participants with an average age of 46.0 ± 12.9 years old. The average compliance rate of participants was 96%, and none had below 80%.

**Figure 8:**
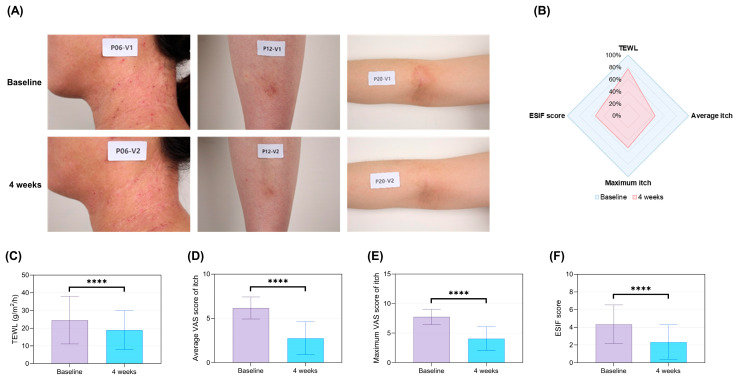
Four weeks of topical DualPep-ATO application significantly reduces itch severity, TEWL, and overall clinical severity. (**A**) Representative clinical photographs of affected skin areas (neck, lower leg, and antecubital fossa) at baseline and after 4 weeks of treatment. (**B**) Radar chart summarizing the percentage reduction from baseline to week 4 for each clinical parameter. Quantitative evaluation of (**C**) TEWL, (**D**) average itch VAS score, (**E**) maximum itch VAS score, and (**F**) ESIF score at baseline and week 4. Data are presented as mean ± SD (n = 21 subjects). **** *p* < 0.001 vs. baseline. TEWL, transepidermal water loss; VAS, Visual Analogue Scale; ESIF, Erythema, Scaling, Induration, and Fissuring.

Visually, the 4-week topical application of DualPep-ATO led to visible within-subject improvements in skin appearance, including reduced erythema, dryness, and scaling in itchy areas (Figure 8A). Radar chart analysis (Figure 8B) demonstrated consistent improvement across all measured parameters, including transepidermal water loss (TEWL), itch severity, and the Erythema, Scaling, Induration, and Fissuring (ESIF) score.

In detail, quantitative assessment revealed a significant within-subject decrease in TEWL compared with baseline, suggesting partially enhanced skin barrier integrity and improved moisture retention (baseline, 24.6 ± 13.5 g/m^2^/h; 4 weeks, 19.0 ± 11.0 g/m^2^/h; *p* < 0.001, effect size: −1 [95% CI: −1, −1]; Figure 8C).

Both the average and maximum itch scores, measured using the Visual Analogue Scale (VAS), significantly decreased after 4 weeks of DualPep-ATO application, reflecting the product’s soothing and anti-pruritic effects on the skin (average itch: baseline, 6.17 ± 1.24; 4 weeks, 2.76 ± 1.87; *p* < 0.001, effect size: −2.04 [95% CI: −2.79, −1.27]; Figure 8D), (maximum itch: baseline, 7.74 ± 1.30; 4 weeks, 4.07 ± 2.00; *p* < 0.001, effect size: −1 [95% CI: −1, −1]; Figure 8E).

The ESIF average score decreased from 4.33 ± 2.20 at baseline to 2.33 ± 1.96 after 4 weeks (*p* < 0.001, effect size: −1 [95% CI: −1, −1]; Figure 8F).

The subjective evaluation of improvement after 4 weeks revealed that 9 participants rated 1 point (very satisfied), 11 people rated 2 points (satisfied), and one person rated 3 points (no change). No adverse effects were observed.

## 3. Discussion

In recent years, CPPs have emerged as a versatile class of bioactive molecules with applications that extend beyond drug delivery, offering direct immunomodulatory and barrier-restorative effects in cutaneous biology. Importantly, a major limitation in the translational advancement of CPPs has been the disconnect between skin penetration capability and functional biological efficacy under formulation-compatible conditions. Many CPPs demonstrate cellular uptake or enhance cargo delivery to promote functional benefits [18,19,24]. However, few studies have established whether this penetration results in meaningful biological modulation when incorporated into real-world topical formulations. In this study, we demonstrated that DualPep-ATO, a penetration-capable molecule with intrinsic biological activity delivered via a topical formulation, exerts multimodal benefits across in vitro, ex vivo, and exploratory clinical settings.

Epidermal barrier damage and pruritus reinforce each other through the “itch–scratch cycle,” driving symptom chronicity in conditions like eczema, xerosis-associated itch, and inflammatory dermatoses. Mechanistically, barrier impairment—typically driven by the downregulation of filaggrin, loricrin, and involucrin—elevates TEWL and heightens susceptibility to environmental triggers [6,25], while pruritic mediators (e.g., IL-31, histamine, and prostaglandins) simultaneously provoke scratching [26,27]. This process further disrupts the barrier and intensifies inflammation, creating a self-perpetuating loop that continually amplifies pruritic signaling. To interrupt this, DualPep-ATO is hypothesized to play a dual therapeutic mechanism: partially restoring cornified envelope proteins in UVB-damaged tissue to repair the barrier, while concurrently inhibiting β-HEX to suppress mast-cell–associated pruritus. These laboratory findings closely align with the clinical reductions observed in TEWL and VAS itch scores, suggesting that these parallel mechanisms drive meaningful symptomatic improvement. Nevertheless, further specialized mechanistic studies are warranted to fully validate these interconnected pathways.

Inflammation represents another central driver of skin barrier decline and pruritus. Activated macrophages and keratinocytes can produce excessive amounts of IL-6, IL-1β, TNF-α, PGE-2, and NO, amplifying tissue damage and altering epidermal differentiation [3,4,28]. In this study, DualPep-ATO suppressed these inflammatory mediators in macrophages while correspondingly reducing erythema and pruritus in clinical subjects. These outcomes align with the previous study on DualPep-ATO, in which the agent suppressed inflammatory cytokine expression and release in TNF-α/IFN-γ-treated human keratinocytes [19]. Together, these results suggest that DualPep-ATO might mitigate both immune- and mast-cell–driven inflammation through the downregulation of cytokine and mediator signaling. However, comprehensive mechanistic studies are required to verify this hypothesis.

Importantly, the preliminary clinical observations in this study indicate favorable tolerability and high participant satisfaction, with no adverse reactions reported throughout the application period. This contrasts with limitations associated with conventional therapies such as topical corticosteroids or calcineurin inhibitors, which may induce skin atrophy, telangiectasia, burning sensations, or increased infection risk when used chronically [6]. However, cautions are warranted when interpreting our results and contrasting them with other therapeutic agents. Despite DualPep-ATO exhibiting some statistical significance when compared with comparator agents, like PVA and HC, these comparisons are context-specific (dose, model, endpoint-dependent) and do not imply clinical superiority over approved pharmacologic agents. Although DualPep-ATO is not intended to replace pharmacological treatments, its multimodal mechanism—lowering inflammatory mediators, reducing itch, and restoring epidermal differentiation markers—suggests value as a complementary or maintenance therapy, especially for individuals requiring long-term skin barrier support.

Despite the multi-layered study design, several limitations should be acknowledged. The clinical study was an open-label trial without a placebo or vehicle-only control group, limiting the ability to exclude placebo effects or distinguish the specific efficacy of DualPep-ATO from the moisturizing effects of the cream vehicle. In addition, the small sample size, short 4-week duration, and clinical heterogeneity of participants with conditions such as dry skin, eczema, and folliculitis limit the generalizability of the findings and prevent assessment of long-term efficacy, relapse rates, or comparisons with established pharmacological treatments.

Regarding laboratory experiments, the reference agents were included only for biological context and do not imply clinical equivalence. Additionally, the acute UVB-induced damage model and use of macrophages do not fully reflect the pathological or inflammatory background in chronic barrier diseases. While barrier restoration was inferred from TEWL and filaggrin, loricrin, and involucrin expression, comprehensive repair cannot be confirmed without evaluating epidermal lipids, tight junction proteins, and functional permeability. Similarly, the β-HEX assay demonstrates suppressed mast cell degranulation but does not establish direct anti-pruritic activity or interference with upstream IgE signaling. Mechanistic validation remained limited, as key pathways such as NF-κB, STAT3, mast cell activation, cytokine transcription networks, and barrier-related differentiation pathways were not experimentally confirmed. Finally, Western blot validation was limited by tissue availability, and the lack of an ex vivo vehicle control prevents definitive attribution of the effects to the peptide alone.

To address these limitations, future research should transition toward rigorous, large-scale randomized, placebo-controlled trials with extended follow-ups to validate clinical efficacy. Mechanistically, future studies must employ advanced proteomic and biophysical assays to map specific binding profiles and signaling cascades. Integrating appropriate vehicle controls in ex vivo setups and comprehensively analyzing lipidomic and structural parameters will be essential to fully resolve the peptide’s mode of action and accelerate its translational development.

## 4. Materials and Methods

### 4.1. Test Product

The test product, DualPep-ATO, was identified using the IDDT platform (Remedi Co., Ltd., Incheon, Republic of Korea), an AI-based deep learning system trained on a high-quality peptide database to discover human-derived peptides with enhanced cell-penetrating properties [19]. It consists of the amino acid sequence LEKHSGKRR. The peptide was synthesized using standard fluorenylmethoxycarbonyl (Fmoc) solid-phase peptide synthesis (SPPS) on 2-chloro trityl chloride resin with protected amino acids. Following completion of chain assembly, the Fmoc protecting group was removed, and palmitoylation was performed by conjugation with palmitic acid. The crude peptide was purified using a C18 reverse-phase column with a linear water–acetonitrile gradient containing 0.1% trifluoroacetic acid (Sigma-Aldrich, Darmstadt, Germany). Peptide purity (>98%) and molecular weight (1110.27 Da) were verified by analytical high-performance liquid chromatography and liquid chromatography–mass spectrometry prior to formulation (Figures S1 and S2). To minimize manufacturing variability, all experiments were conducted using a single production batch of DualPep-ATO.

DualPep-ATO was then incorporated into 2 formulations:

- A transparent liquid form for the in vitro study (solvents: water, sodium phosphate dibasic, sodium phosphate monobasic).
- An opaque white cream form containing 0.01% concentration of DualPep-ATO for the ex vivo and clinical studies (Remedi Co., Ltd., Incheon, Republic of Korea).

Each formulation’s ingredients are listed in Table 1 and Table 2.

### 4.2. In Vitro Study

#### 4.2.1. Cell Culture

RBL-2H3 cells (American Type Culture Collection, Manassas, VA, USA), which exhibit characteristics of both mast cells and basophils and are widely used to assess mast cell–associated degranulation in allergy research, were selected for anti-pruritus evaluation [29]. Meanwhile, the Raw 264.7 cells (American Type Culture Collection, Manassas, VA, USA) is frequently used to evaluate macrophage-mediated inflammatory responses [30]. In our study, both cell types were cultured in Dulbecco’s Modified Eagle’s Medium (DMEM; Hyclone, Logan, UT, USA) supplemented with 10% fetal bovine serum (Gibco, Waltham, MA, USA) and 1% penicillin-streptomycin. HEKn (Thermo Fisher Scientific, Waltham, MA, USA) were cultured in EpiLife™ Medium (Gibco) supplemented with Human Keratinocyte Growth Supplement (Gibco) under standard conditions of 37 °C and 5% CO_2_.

#### 4.2.2. Cell Viability and Proliferation Assessment

Cells were seeded at a density of 5 × 10^4^ cells per well in 96-well plates. Upon reaching approximately 80% confluence, they were treated with various concentrations of DualPep-ATO (0.8, 4, 20, 100 µg/mL) and incubated for 24 h. After treatment, WST substrate solution (CCK-8; Dojindo, Rockville, MD, USA) was added to each well, and the plates were incubated at 37 °C for an additional 2 h. Absorbance was then measured at 450 nm using a microplate reader (VARIOSKAN LUX; Thermo Fisher Scientific). The optical density (OD) values were blank-corrected and normalized to the negative control, with higher absorbance indicating greater cell viability. L-NMMA (25 µM; Sigma-Aldrich, M7033), ketotifen (12.5 µg/mL; Sigma-Aldrich, K2628), and EGCG (10 µM; Sigma-Aldrich, E4143) were used as positive controls in Raw 264.7, RBL-2H3, and HEKn cells, respectively. These agents were selected based on their well-established biological activities relevant to each assay system. Specifically, L-NMMA was used in the anti-inflammatory assay due to its inhibitory effect on NO production [31]; ketotifen was included in the anti-allergic assay as a well-characterized mast cell stabilizer [32]; and EGCG was applied in the barrier-enhancing assay based on previous reports demonstrating its effects on keratinocyte differentiation and skin barrier function [33].

#### 4.2.3. β-HEX Assay

To evaluate β-HEX release, RBL-2H3 cells were seeded at a density of 2 × 10^5^ cells per well in 24-well plates. Cells were sensitized by incubation in serum-free DMEM supplemented with DNP-IgE (100 ng/mL; Sigma-Aldrich, St. Louis, MO, USA) for 24 h to induce an allergic response. Cells were then washed twice with PIPES buffer and incubated in PIPES buffer containing 4 mM MgCl_2_, 5.6 mM glucose, and 0.1% bovine serum albumin (BSA). Cells were then treated with DualPep-ATO (4, 20, or 100 µg/mL) or ketotifen (12.5 µg/mL) as a positive control for 1 h, followed by stimulation with DNP-BSA (100 ng/mL) for an additional 1 h to induce IgE-dependent activation of RBL-2H3 cells. The reaction was terminated by placing the plates on ice for 10 min. A 20 μL aliquot of supernatant was mixed with 80 μL of 1 mM 4-pNitrophenyl-N-acetyl-b-D-glucosaminide (Sigma-Aldrich; N9376) in a 96-well plate and incubated at 37 °C for 1 h. The reaction was stopped by adding 200 µL of stop solution [0.1 M NaHCO_3_ (T&I, BSB-8098, Seoul, Republic of Korea) + 0.1 M Na_2_CO_3_ (Sigma-Aldrich, 223530)], and optical density (OD) was measured at 405 nm using the VARIOSKAN LUX reader. Higher values indicated increased β-HEX release.

#### 4.2.4. NO Assay

NO, an inflammatory mediator produced by activated macrophages, was quantified to evaluate anti-inflammatory activity of DualPep-ATO similar to our previous study [34]. Raw 264.7 cells were seeded at a density of 1 × 10^6^ cells per well in 12-well plates and cultured to approximately 80% confluence. Inflammation was induced by incubation in serum-free DMEM containing LPS (1 μg/mL; Sigma-Aldrich) for 24 h, followed by treatment with DualPep-ATO (4, 20, or 100 μg/mL) or L-NMMA (25 μM) as a positive control for an additional 24 h. Culture supernatants were collected, centrifuged at 2000× *g* for 10 min, and mixed 1:1 with Griess reagent (Sigma-Aldrich) for subsequent analysis. After incubation at room temperature, OD was measured at 540 nm using the VARIOSKAN LUX reader. NO concentration was determined from a NaNO_2_ standard curve, with higher absorbance indicating greater NO production.

#### 4.2.5. Enzyme-Linked Immunosorbent Assay (ELISA)

We next evaluated the production of inflammatory cytokines TNF-α, IL-1β, and PGE-2 to assess the anti-inflammatory activity of DualPep-ATO similar to our previous study [35]. Specifically, Raw 264.7 cells (1 × 10^6^ cells/well) were seeded in 6-well plates. Upon reaching 80% confluence, LPS (1 μg/mL) was applied to induce inflammation, followed by treatment with DualPep-ATO (4, 20, and 100 µg/mL), or L-NMMA (25 μM), positive control. After 24 h of incubation, culture media were harvested and centrifuged at 2000× *g* for 10 min to remove cellular debris. The clarified supernatants were subsequently analyzed using ELISAs, including the Mouse TNF-α SimpleStep ELISA kit (ab208348; Abcam, Cambridge, UK), Mouse IL-1β SimpleStep ELISA kit (ab197742; Abcam), and Mouse PGE-2 ELISA kit (MBS266212; MyBioSource, San Diego, CA, USA).

HEKn cells were plated in 6-well culture plates at a density of 5 × 10^4^ cells per well. Next, we irradiated HEKn cells with UVB using a similar protocol to our previous study [34]. Upon reaching approximately 80% confluence, the cells were subjected to ultraviolet B (UVB) irradiation at a dose of 10 mJ/cm^2^ using a UV cross-linker (BLX 312; Vilber Lourmat, Marne-la-Vallée, France; emission range 280–320 nm). This irradiation dose was chosen based on prior reports indicating reliable induction of cellular stress and epidermal barrier impairment without inducing excessive cytotoxicity [36,37]. To ensure dose accuracy and reproducibility, UVB output was calibrated before each experiment using a UV radiometer. UV intensity was measured at the level of the cell monolayer with a UV meter (UV-340A, LT Lutron, Coopersburg, PA, USA; band pass 260–390 nm), and the delivered UVB dose (mJ/cm^2^) was calculated by multiplying the measured irradiance (mW/cm^2^) by the exposure duration (seconds), which was typically under 10 s for the specified dose. Following UVB exposure, cells were treated with DualPep-ATO (4, 20, or 100 µg/mL) or EGCG (10 µM) as a positive control. After 24 h of treatment, cells were washed with cold phosphate-buffered saline (PBS) and lysed using PRO-PREP™ Protein Extraction Solution (C/T) (17081, iNtRON Biotechnology Inc., Seongnam, Republic of Korea). Cell lysates were collected, clarified by centrifugation, and the resulting supernatants were subjected to ELISA analysis using the Human Filaggrin ELISA kit (MBS7251319; MyBioSource), Human Loricrin ELISA kit (MBS2019763; MyBioSource), and Human Involucrin ELISA kit (MBS2703191; MyBioSource).

The assays were performed according to the provided protocols, and OD was measured using the VARIOSKAN LUX reader, with higher values indicating greater protein levels.

### 4.3. Ex Vivo Study

#### 4.3.1. Human Skin Tissue Culture

Human skin tissue experiments were approved by the Global Medical Research Center Institutional Review Board (IRB No. GIRB-25605-OB). Skin samples from three independent donors were defatted and washed with PBS to remove residual contaminants. The tissues were subsequently sectioned into 1 × 1 cm^2^ specimens and exposed to UVB irradiation using a UV cross-linker (BLX 312; Vilber Lourmat) at a wavelength of 312 nm with a total dose of 300 mJ/cm^2^ to induce barrier disruption [38,39]. The emission spectrum (280–320 nm) and output intensity were continuously monitored to ensure UVB-specific exposure. Following UVB irradiation, tissue samples were treated with a cream formulation containing 0.01% DualPep-ATO, with HC 1% (Taekgeuk Pharm. Co., Ltd., Seoul, Republic of Korea) and 0.15% PVA (Sama Pharm. Co., Ltd., Seoul, Republic of Korea) used as comparator treatments. The tissues were then maintained at 37 °C in a humidified atmosphere containing 5% CO_2_ using a semi-solid culture medium for 24 h. UVB irradiation, topical treatment, tissue incubation, and culture medium replacement were repeated every 24 h for a total of 72 h (three cycles). The culture medium matrix was prepared as described in our previous study [40].

#### 4.3.2. ELISA

After 72 h of test compound application, the collected tissues were homogenized in PRO-PREP Protein Extraction Solution (iNtRON Biotechnology, Seongnam, Republic of Korea) using a TissueLyser II (Qiagen, Hilden, Germany). The homogenates were centrifuged at 2000× *g* for 10 min, and the resulting supernatants, with tissue debris excluded, were collected for subsequent ELISA analysis. To partially evaluate skin barrier-related proteins, the levels of filaggrin, loricrin, and involucrin were quantified using the same ELISA kits and procedures described for the HEKn experiments (Section 4.2.5). To assess anti-inflammatory effects, the concentrations of TNF-α, IL-1β, and IL-6 were measured using the Human TNF-α ELISA Kit (KHC3014; Invitrogen, Waltham, MA, USA), Human IL-1β ELISA Kit High Sensitivity (A323168; Antibodies.com, Cambridge, UK), and Human IL-6 ELISA Kit High Sensitivity (ab46042; Abcam), respectively, according to the manufacturers’ instructions.

#### 4.3.3. IF Assay

After 72 h of treatment, human skin tissues were collected and embedded in OCT to prepare 4 μm frozen sections using a cryostat microtome (Leica Biosystems, Nussloch, Germany). Sections were mounted on silane-coated slides, the OCT compound was removed, and samples were incubated with primary antibodies (filaggrin: ab81468, dilution, 1:500; loricrin: ab85679, dilution, 1:200; involucrin: ab68, dilution, 1:100; Abcam). After washing, sections were treated with FITC-conjugated secondary antibody (Goat pAb to Rb IgG-FITC: ab6717, dilution, 1:200; Abcam) and counterstained with 4′,6-Diamidino-2-Phenylindole (DAPI, VECTASHIELD^®^ Mounting Medium with DAPI; Vector Laboratories, Newark, CA, USA). Images were captured at 400× magnification using a confocal laser scanning microscope (LSM700; Zeiss, Oberkochen, Germany), and fluorescence intensity was quantified using ImageJ software version 1.53 (NIH, Bethesda, MD, USA).

All experiments were conducted in three independent replicates to ensure reproducibility of the results.

### 4.4. Clinical Study

#### 4.4.1. Clinical Study Design and Participants

This exploratory, hypothesis-generating, open-label clinical trial was conducted at the Global Medical Research Center, Seoul, Republic of Korea, with approval from Global Medical Research Center Institutional Review Board (IRB No. GIRB-25620-OL) and adherence to the Declaration of Helsinki. Participants aged 19–70 years who were diagnosed with skin conditions causing pruritus (e.g., dry skin, eczema, folliculitis) and impaired skin barrier function (baseline TEWL ≥ 12 g/m^2^/h) by a board-certified family medicine specialist (A.M.) were enrolled. Exclusion criteria included individuals with active dermatologic conditions (except atopic dermatitis) requiring treatment; those who had used antibiotics, steroids, immunosuppressants, antihistamines, retinoids, or received phototherapy within 4 weeks of screening (unless non-interfering as determined by the investigator); participants with intentionally damaged skin, known cosmetic allergies, or enrolled in other studies; pregnant or breastfeeding women, or those not using adequate contraception; and participants unable to attend follow-up visits or deemed unsuitable by the investigator.

Sample Size: A minimum of 20 participants were recruited, in accordance with the Regulations on Functional Cosmetics Evaluation (MFDS No. 2021-55) [41] and the Guideline for Test Methods for Cosmetic Labeling and Advertising Verification (MFDS, 2018) [42], ensuring statistical reliability for comparative analysis.

Participants were withdrawn if they voluntarily discontinued, developed skin-related adverse reactions, had excessive UV exposure, engaged in behaviors that could compromise study integrity (e.g., excessive alcohol consumption or smoking), or faced personal circumstances preventing continuation.

#### 4.4.2. Study Procedures

At Visit 1, participants were screened for eligibility, provided written informed consent, and assigned unique identification codes. After a 30 min acclimation period under controlled conditions (20–24 °C, 45–55% humidity), baseline skin measurements were taken using calibrated devices. The VAS and ESIF were assessed by a board-certified family medicine specialist (A.M.). Participants were then given a cream containing 0.01% DualPep-ATO and a compliance diary for home use. The product was applied twice daily (morning and evening) to the cleansed skin area, using one fingertip unit—enough to cover both palms. It was evenly massaged until fully absorbed, for a total of 56 applications over 4 weeks.

At Visit 2, the same acclimation and assessment procedures were repeated, including VAS and ESIF evaluations. Participants also completed a satisfaction questionnaire, and both the test product and compliance diary were collected to assess adherence. Any adverse events or concomitant medication use were monitored and documented at each visit to ensure participant safety and data reliability.

#### 4.4.3. Clinical Improvement Efficacy Assessment

##### Objective Measurement Items

TEWL was measured using a Tewameter TM300 (Courage + Khazaka Electronic, Cologne, Germany). After stabilization, the mean of the final three readings from each site was used for analysis. Results were expressed in g/m^2^/h, with lower values indicating improved skin barrier function.

Photographic documentation was performed using a Canon EOS 650D digital camera (Canon Inc., Tokyo, Japan) to capture designated skin areas (e.g., 3 cm below the antecubital fossa or 5 cm below the popliteal fossa). All images were taken under controlled conditions (same location, lighting, and camera settings) following a standardized protocol to ensure consistency and facilitate accurate comparison of skin condition over time.

##### Participants’ Pruritus Assessment (VAS)

Participants assessed their average and maximum itch intensity over the past 24 h using a 10 cm VAS tool. They marked their perceived itch level on the line, and the distance from the starting point was measured in centimeters. Pruritus improvement was determined by comparing VAS scores over time, with shorter lengths indicating greater relief (Table 3).

##### Expert Visual Assessment

A board-certified family medicine specialist (A.M.) evaluated the severity of 4 eczema characteristics using the ESIF scale (Table 4). The total score was calculated as the sum of the 4 parameters, ranging from 0 to 12.

##### Participant Satisfaction Assessment

At each visit, participants evaluated their satisfaction with the test site condition relative to baseline. The assessment was performed using a 5-point scale (Table 5).

##### Safety Evaluation and Compliance Assessment

The incidence rate was calculated and incorporated into the overall safety assessment of the test product. Data from participants who applied the test product less than 80% of the prescribed total applications were excluded from the final analysis.

### 4.5. Statistical Analysis

All statistical analyses were performed using IBM SPSS Statistics version 27.0, and graphing was conducted using GraphPad Prism version 10.2.2. The significance of differences before and after product use was evaluated at a 5% significance level (*p* < 0.05). For the clinical study, data normality was first assessed, after which appropriate tests were applied. Specifically, to compare paired measurements within each group, a parametric paired samples *t*-test was used for normally distributed data, whereas the nonparametric Wilcoxon signed-rank test was employed for non-normally distributed data. The effect size was calculated using Cohen’s d (for paired *t*-test) and rank biserial (for Wilcoxon signed-rank test). Regarding the laboratory studies, the Mann–Whitney U test was used to compare values between groups.

## 5. Conclusions

In summary, our integrated in vitro, ex vivo, and exploratory clinical analyses suggest that DualPep-ATO may attenuate inflammatory pathway signaling, reduce pruritic responses, and support restoration of epidermal barrier proteins, consistent with a multi-target mode of action that remains to be defined. Given the exploratory design and limited sample size of the clinical component, these findings are hypothesis-generating. Nonetheless, their convergence across independent model systems supports further investigation of DualPep-ATO as a topical peptide candidate for barrier-impaired, pruritic conditions, pending randomized, vehicle-controlled trials.

## Figures and Tables

**Table 1:** Ingredients in DualPep-ATO (transparent liquid form).

No.	Ingredient Name
1	Water
2	Sodium phosphate dibasic
3	Sodium phosphate monobasic
4	DualPep-ATO

**Table 2:** Ingredients in DualPep-ATO (opaque cream form).

No.	Ingredient Name
1	Water
2	Glycerin
3	Caprylic/Capric Triglyceride
4	Panthenol
5	Dicaprylyl Carbonate
6	Butyrospermum parkII (Shea) Butter
7	Theobroma Cacao (Cocoa) Seed Butter
8	1,2-Hexanediol
9	Sorbitan Olivate
10	Butylene Glycol
11	Simmondsia Chinensis (Jojoba) Seed Oil
12	Cetearyl Olivate
13	Palmitic Acid
14	Centella Asiatica Extract
15	Diisostearyl Malate
16	Ceramide NP
17	DualPep-ATO
18	Cetyl Alcohol
19	Stearic Acid
20	Hydrogenated Lecithin
21	Carbomer
22	Phytosterols
23	Ammonium Acryloyldimethyltaurate/VP Copolymer
24	Arginine
25	Acrylates/C10-30 Alkyl Acrylate Crosspolymer
26	Allantoin
27	polyglyceryl-3 Methylglucose Distearate
28	Sodium Phytate
29	Sodium Hyaluronate

**Table 3:** The Degree of Itching (VAS).

Score	Degree of Itching
0	No itching
≥1 to <3	Mild itching
≥3 to <7	Moderate itching
≥7 to <9	Severe itching
10	Very severe itching

**Table 4:** Erythema, Scaling, Induration, and Fissuring (ESIF) scale.

Parameter	0 Point	1 Point	2 Points	3 Points
Erythema	None	Light pink	Red (not dark red)	Deep/dark red to purple
Scale	None	Fine scaling	Diffuse, thick scaling	Very thick scaling; all lesions covered
Induration	None	Slightly elevated lesion (~0.5 mm)	Clearly indurated (~0.75 mm elevation)	At least 1 mm elevation
Fissure	None	Several superficial fissures	Multiple moderate-depth fissures	Numerous and deep fissures

**Table 5:** Participant Satisfaction Criteria.

Score	Category	Criteria
1	Very satisfied	Significant overall improvement in itching
2	Satisfied	Noticeable overall improvement in itching
3	No change	No difference compared to before application
4	Dissatisfied	Overall worsening of itching
5	Very dissatisfied	Significant overall worsening of itching

## Data Availability

The original contributions presented in this study are included in the article/Supplementary Materials. Further inquiries can be directed to the corresponding author.

## Supplementary Materials

The following supporting information can be downloaded at: https://www.mdpi.com/article/10.3390/ijms27146357/s1.

## Abbreviations

The following abbreviations are used in this manuscript:

β-HEX	Beta-Hexosaminidase
CO_2_	Carbon Dioxide
CPPs	Cell-Penetrating Peptides
DAPI	4′,6-Diamidino-2-Phenylindole
DMEM	Dulbecco’s Modified Eagle’s Medium
DNP-BSA	2,4-Dinitrophenylated Bovine Serum Albumin
DNP-IgE	Anti-Dinitrophenyl Immunoglobulin E
EGCG	Epigallocatechin Gallate
ELISA	Enzyme-Linked Immunosorbent Assay
ESIF	Eczema Severity Index for Itch and Flaking Scale
Fmoc	Fluorenylmethoxycarbonyl
HC	Hydrocortisone
HEKn	Human Epidermal Keratinocytes
IDDT	Intra-Dermal Delivery Technology
IF	Immunofluorescence
IL	Interleukin
L-NMMA	NG-methyl-L-arginine acetate salt
LPS	Lipopolysaccharide
NF-κB	Nuclear Factor Kappa B
NO	Nitric Oxide
OD	Optical Density
PGE-2	Prostaglandin E2
PVA	Prednisolone Valeroacetate
SD	Standard Deviation
SP	Speckled Protein
SPPS	Solid-Phase Peptide Synthesis
STAT3	Signal Transducer and Activator of Transcription 3
TEWL	Transepidermal Water Loss
TNF-α	Tumor Necrosis Factor-alpha
UVB	Ultraviolet B
VAS	Visual Analogue Scale
