# Supplementary material for: Translational Assessment of a Cell-Penetrating Peptide Topical Formulation for Repairing Barrier Dysfunction in Human Skin
Source: Int J Mol Sci. 2026 Jul 17;27(14):6357. doi: 10.3390/ijms27146357 (PMC13410421; doi:10.3390/ijms27146357)
Supplement: Supplementary file 1 [file ijms-27-06357-s001.zip › ijms-4349723-supplementary.pdf]

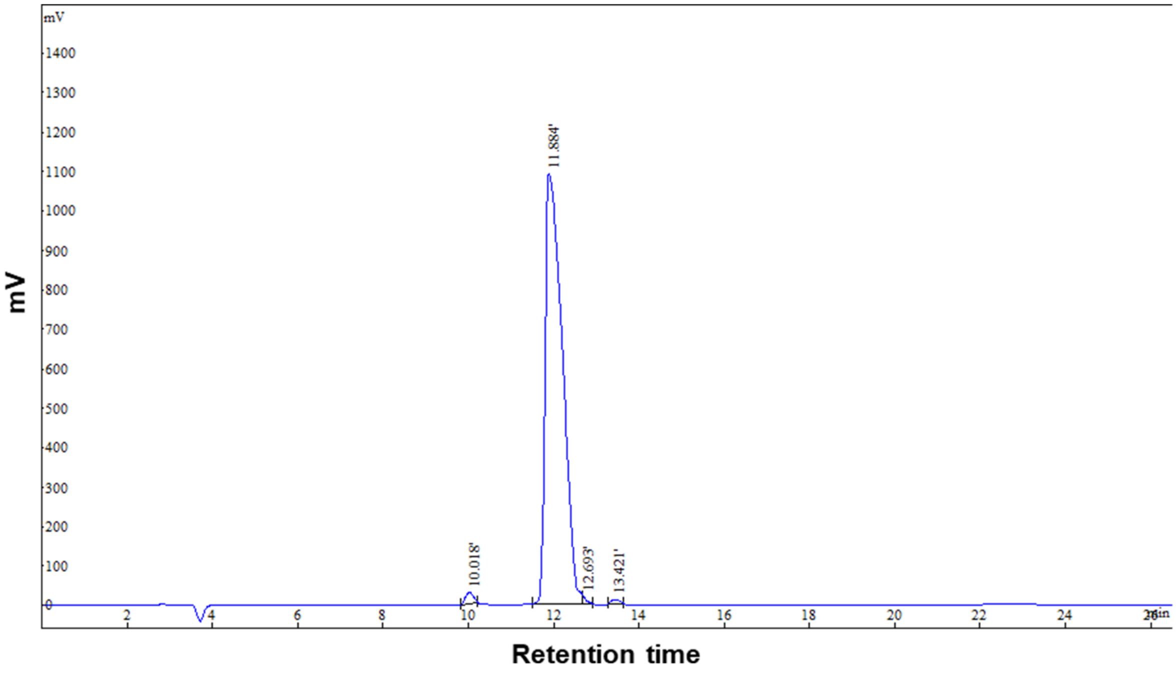

Peak	Retention Time (min)	Area	Purity (% Area)	Height
1	10.018	346456	1.158	27133
2	11.884	29346813	98.09	1089067
3	12.693	91262	0.305	17070
4	13.421	132429	0.443	9516

Figure S1. High-performance liquid chromatography analysis showing high peptide purity (> 98%).

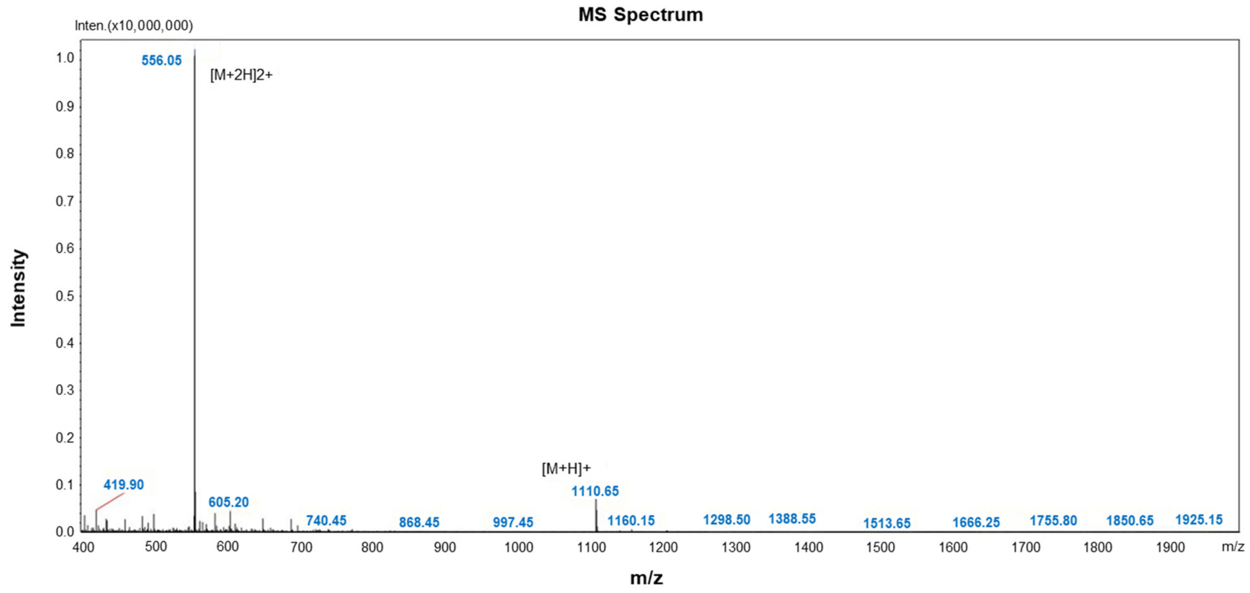

Figure S2. Liquid chromatography–mass spectrometry analysis of the molecular weight of DualPEP-ATO.
